# Can the Childhood Physical Activity Questionnaire Be Used to Identify Physical Activity Levels in Children With Asthma?

**DOI:** 10.3389/fped.2021.726695

**Published:** 2021-10-06

**Authors:** Mattienne R. van der Kamp, Bram W. Nieuwdorp, Boony J. Thio, Monique Tabak, Arvid W. A. Kamps, Hermie J. Hermens, Jean M. M. Driessen

**Affiliations:** ^1^Department of Biomedical Signals and Systems, University of Twente, Enschede, Netherlands; ^2^Department of Pediatrics, Medisch Spectrum Twente, Enschede, Netherlands; ^3^Roessingh Research and Development, Enschede, Netherlands; ^4^Department of Pediatrics, Martini Ziekenhuis, Groningen, Netherlands; ^5^Independent Researcher, Groningen, Netherlands; ^6^Department of Sports Medicine, Ziekenhuis Tjongerschans, Heerenveen, Netherlands

**Keywords:** asthma, child, exercise, accelerometry, self-report, wearable electronic devices

## Abstract

**Objective:** Children with asthma who are physically active have a better quality of life, emphasizing the importance of activity monitoring and promotion in daily life. The validity of self-reported activity measurements has been questioned in pediatric populations. In this study, we aim to compare the Physical Activity Questionnaire for Children (PAQ-C) with objectively measured PA using accelerometry.

**Design:** In this comparison study, the pooled dataset of two cross-sectional studies was used, which prospectively home-monitored PA using the alternative self-report PAQ-C questionnaire as well as with the criterion standard accelerometry (Actigraph wGT3X-BT and GT1M).

**Participants:**Ninety children with pediatrician-diagnosed asthma participated in the study.

**Main Outcome Measures:**Correlation coefficients were calculated to determine the relation between the PAQ-C and accelerometer data. The predictive value of the PAQ-C in differentiating between achieving and failing the recommended daily level of moderate-to-vigorous activity (MVPA) was evaluated with receiver operator characteristic (ROC) analysis.

**Results:** The results showed weak to moderate correlations of the PAQ-C with the accelerometer data (*r* = 0.29–0.47). A PAQ-C cutoff of 3.09 showed the best performance on predicting whether the recommended level of MVPA was achieved. With this cutoff, 21 of the 39 children that did achieve their daily MVPA level (53.8% sensitivity) and 33 of the 46 children that did fail their daily MVPA level (71.7% specificity) were correctly classified. A PAQ-C score of 3.5 revealed a negative predictive value of 100% for assessing physical inactivity.

**Conclusion:** This study revealed a weak relation between the PAQ-C and PA assessed with accelerometry. However, a PAQ-C score of 3.5 or higher might be used as a low-cost and easy-to-use PA screening tool for ruling out physical inactivity in a portion of the pediatric asthma population.

**Clinical Trial Registration:** Netherlands Trial Register: Trial NL6087.

## Introduction

Children with asthma who are physically active in daily life have a better quality of life (1). Moreover, physical activity (PA) is associated with healthy neuromuscular and cardiovascular development, reduced risk for chronic diseases, and improved psychological wellbeing among children (2). In children with asthma, being physically active brings extra benefits by reducing airway inflammation and by mediating the relation between asthma and obesity (3, 4).

The goal of pediatric asthma treatment is to enable children with asthma to be as active as their peers. Therefore, current asthma guidelines recommend to follow the general public health guidance of an hour of moderate-to-vigorous intensity physical activity (MVPA) per day (5, 6). The Global Initiative for Asthma (GINA) further recommends that children with asthma should be encouraged to exercise and participate in sports, as regular aerobic exercise improves the management of asthma symptoms, lung function, and experiencing other health benefits (7, 8).

In order to comply with these guidelines, the care professional should be able to intervene in case of physical inactivity. To facilitate the identification of inactive children, there is a need for an adequate and easy-to-use PA assessment tool. A wide range of methods have been used to quantify PA behavior in children with asthma. These methods include both subjective PA self-reports in the form of PA diaries or PA questionnaires and objective measures such as direct observation, heart rate monitoring, pedometers, and accelerometry (9, 10).

Self-reported activity measures are a low-cost and easy-to-use tool for children to reflect their PA experiences; however, these measures are susceptible to all kinds of prepossessions, such as interpretation bias, recall bias, and questionnaire design bias (11, 12). The validity and reliability of self-reported activity measurements have been questioned in pediatric populations (13, 14). Furthermore, there is a general consensus for a consistent overestimation of PA using self-report instruments (15). Trost et al. (16) indicated this issue by stating, “Precise measures of habitual physical activity are a necessity in studies designed to document the frequency and distribution of physical activity in defined population groups”.

Accelerometry, on the other hand, is an often used and objective method for assessing PA behavior in asthmatic children (17–19). Moreover, accelerometer-based PA monitoring devices are mostly unobtrusive and well-validated against other measures such as direct observation or oxygen consumption (VO_2_ or doubly labeled water) (20, 21). This makes accelerometry an adequate choice to accurately capture children's highly transitory and intermittent PA patterns.

Accurate, reliable, and feasible methods for monitoring of the PA behavior in children continue to be a research priority (10, 16, 22). In this study, we compare the self-reported Physical Activity Questionnaire for Children (PAQ-C) score with objectively measured PA using accelerometry to (1) investigate the relation between subjective and objectively assessed PA in children with asthma and (2) investigate the predictive value of the PAQ-C in assessing physical inactivity and recommended levels of MVPA.

## Methods

### Study Design

This study used a pooled dataset of two cross-sectional studies: the WEARCON study (23), which investigated the use of wearable home-monitoring devices to assess asthma control, and the ACCOR study, which investigated the association between asthma control and cortisol levels.

In both studies, daily PA of all participating asthmatic children were prospectively monitored both subjectively using self-report PAQ-C questionnaire as well as objectively using accelerometry. This resemblance in study design combined with the similar participant selection and the required sample size allowed the opportunity to investigate the pooling of these datasets (24).

Both studies were conducted according to the Helsinki declaration, approved by the medical ethics committee (ACCOR: MEC Martini hospital 2016-083 and WEARCON: METC Twente P16-27). The ethical committee (MEC Martini hospital 2018-129) approved the pooling of the data from both studies for the aim of this study and all participants (children and parents) provided their informed consent.

### Sample Size

The required sample size for the correlation between self-reported and accelerometer-based activity was determined using power analysis. The power analysis was conducted in G-POWER using an alpha of 0.05, a power of 0.80, and a medium effect size (ρ = 0.3) for a two-tailed test, which was chosen slightly below previous reported results (25), to ensure power. Based on the aforementioned assumptions, the required sample size was calculated to be 84.

### Subjects

Sixty children aged between 4 and 14 years old of the Medisch Spectrum Twente hospital, Enschede and 30 children of the Martini hospital, Groningen, The Netherlands were included in, respectively, the WEARCON and ACCOR study using consecutive sampling. These children were diagnosed with asthma by the pediatric pulmonologist according to the GINA guidelines (7) and eligible for the analysis of this study. Exclusion criteria were other (chronic) conditions interfering with daily activity, such as psychomotor retardation, injuries, neuromuscular disorders, and chronic fatigue syndrome.

### Baseline Characteristics

Demographic characteristics, such as age, body mass index (BMI) *z*-scores, gender, allergic rhinitis diagnosis, and medication use, were obtained from the electronic patient record. Lung function tests were performed to assess baseline spirometry values [i.e., forced expiratory volume in 1 s (FEV_1_)]. The children completed a childhood asthma control test (C-ACT) questionnaire with their parents (26).

### Objectively Measured Physical Activity: Accelerometry

The Actigraph wGT3X-BT^a^ was used in the WEARCON study and the Actigraph GT1M^a^ was used in the ACCOR study to objectively assess raw accelerometer counts. The devices did not show interpretable data to the subjects to prevent any influence, and data were stored anonymously on the device.

The children and parents received the Actigraph and instruction materials, and were instructed by the researcher. The subjects wore the activity tracker for 14 (WEARCON) or 7 (ACCOR) consecutive days in representative school weeks, without (bank) holidays, reflecting the subjects' average habitual activities (27). Subjects within the WEARCON study were instructed to attach the tracker at the wrist location of the non-dominant hand. Subjects of the ACCOR study attached the tracker with an elastic band to the non-dominant hip. In both studies, subjects were instructed to remove the activity tracker before activities involving water (such as showering or swimming).

### Subjectively Measured PA: PAQ-C

The level of daily PA was recorded using a validated questionnaire, the PAQ-C. The PAQ-C was originally developed for healthy children between the ages of 8 and 14 to represent activities performed in the past 7 days (28). This questionnaire was chosen as no specific activity questionnaires for a pediatric asthma population were available and because the PAQ-C was found to be most reliable, well validated, and clinically used as pediatric activity questionnaire (25, 29–31). The questionnaire was translated into Dutch and validated for children (32). The questionnaire consists of nine questions. Scores per question range from one to five, with the highest score indicating that the activity has often been performed. The final score is determined by first determining the average score per question. These scores are then added and divided by the number of questions, namely, nine. A final score of 1 means low PA, and a score of 5 means high PA.

The %MVPA was estimated based on Saint-Maurice et al. (33). They constructed a calibrated PAQ tool that estimates %MVPA based on the PAQ-C score, while adjusting for age and gender. The model [%MVPA = 14.56 – (sex^*^0.98) – (0.84^*^age) + (1.01^*^PAQ)] explained 40% of the variance in their data and is therefore assumed to be a more precise estimate.

### Data Preprocessing and Analysis

Data analysis of the PAQ-C and Actigraph data took place after the study period to prevent outcome bias. The Actigraph data were preprocessed in the Actilife software ^a^. Raw data were downloaded from the devices with an epoch length of 15 s. Wear time was assessed using the algorithm of Troiano et al. (34). Total wear time had to be at least 75% of the expected wear days to be eligible for further preprocessing and analysis. The wrist worn (tri-axial) activity was classified according to the PA classification of Chandler et al. (20); hip worn (uni-axial) activity was classified with the cut-points of Evenson (21).

PA outcome measures yielded the total amount of PA and the number of minutes spent at each of four activity levels (sedentary, light, moderate, and vigorous activity). All activity parameters were averaged per day and, from there, averaged over the total period of home-measured PA.

### Statistical Analysis

Missing data were handled using pairwise deletion. Data of the two datasets were merged together and labeled by the original dataset. Descriptive statistics were used to examine all continuous outcome measures (both from the original datasets as pooled together) and were expressed in means ± standard deviation (SD) for normally distributed variables and with median [interquartile range (IQR)] for non-normal distributed variables. The Shapiro–Wilk test was used to determine whether the variables were normally distributed. Normally distributed data were compared using an independent samples *t*-test at a 95% confidence interval. Non-normally distributed data were compared using a Mann–Whitney *U* test at a 95% confidence interval. Chi-square tests were performed for discontinuous variables and Fisher's exact test was performed for binary variables.

Cronbach's alpha was calculated to test for the internal consistency of the PAQ-C questionnaire. A Cronbach's alpha coefficient of 0.70 was used a cutoff to allow the use of the PAQ-C questionnaire in this pediatric asthma population. In addition, inter-item raw correlation coefficients were calculated to test for uniformity of the reliability as suggested by Clark and Watson (35).

Both Spearman's rho (rounded PAQ-C scores) and Pearson's *R* (non-rounded PAQ-C scores) were calculated to determine the correlation between the activity parameters (total activity, MVPA, and vigorous activity) and the PAQ-C score. Correlation coefficients were interpreted as very weak (0.00–0.19), weak (0.20–0.39), moderate (0.40–0.59), strong (0.60–0.79), and very strong (0.80–1.00).

A receiver operator characteristic (ROC) curve was computed to evaluate the value of the PAQ-C in differentiating between achieving and failing the recommended daily level of MVPA and for assessing physical inactivity (defined as <50 MVPA). The best cut-point was identified using the maximal Youden's index, with the limitation that both sensitivity and specificity should be at least 50%. This cut-point was used to determine relevant diagnostic validity measures, such as sensitivity, specificity, and positive and negative predictive value. The same ROC analysis was performed with the calibrated PAQ score of Saint-Maurice et al. (33).

## Results

Table 1 shows an overview of the baseline characteristics of all children. The total group of children has a median age of 10 years, consisting primarily of boys (77.8%), and the median BMI *z*-score is 0.41. Medication characteristics in this group show that two-thirds of the children used inhaled corticosteroids (ICS) and 12.2% (11/90) used an ICS and long-acting beta-agonist (LABA) combo. The asthma characteristics show that almost three in four of the children had allergic rhinitis, that their average baseline lung function was 90.5% predicted, and that their mean C-ACT symptom score was 21. The age, BMI *z*-score, and the C-ACT scores differed between the two study groups.

**Table 1:** Baseline characteristics.

	**Total (*n* = 90)**	**Children with asthma in WEARCON study (*n* = 60)**	**Children with asthma in ACCOR study (*n* = 30)**	**Significance**
Age (months)	120 (84–132)	114 (72–132)	120 (96–144)	*p* = 0.04
Gender (% male)	77.8%	80.0%	73.3%	n. s. (*p* = 0.59)
BMI *z*-score	0.41 ± 1.46	0.53 ± 1.38	−0.22 ± 1.48	*p* = 0.02
ICS use (%)	66.7%	68.3%	63.3%	n. s. (*p* = 0.81)
ICS+LABA use (%)	12.2%	8.3%	20.0%	n. s. (*p* = 0.35)
Allergic rhinitis (%)	73.8%	72.0%	76.7%	n. s. (*p* = 0.79)
Baseline LF (% predicted)	90.5 ± 11.5%	91.7 ± 11.1%	88.3 ± 12.2%	n. s. (*p* = 0.26)
C-ACT scores, Percentage ≤ 19	21 (17–24), 45.0%	22 (18–25), 35.0%	18 (15–22), 64.5%	*p* < 0.01*, p* < 0.01

*Data are shown as mean ± SD, %, or median (IQR). Non-significant results are indicated as n. s*.

Within the pooled data of both studies, four children had insufficient wear time of their activity tracker, making their activity data not eligible for further analysis. Moreover, one child did not fill in the PAQ-C questionnaire at the end of the activity-monitoring period. The data of the remaining 85 children are shown in Table 2 and were used as input for the linear regression analysis and the calculation of the ROC curve.

**Table 2:** Activity characteristics.

	**Total (*n* = 85)**	**Children with asthma in WEARCON study (*n* = 56)**	**Children with asthma in ACCOR study (*n* = 29)**	**Significance**
Sedentary activity (min/day)	364 (331–416)	351 (328–394)	374 (332–419)	n. s. (*p* = 0.32)
Light activity (min/day)	246 ± 47	238 ± 48	250 ± 47	n. s. (*p* = 0.38)
Moderate activity (min/day)	40 ± 14	39 ± 12	41 ± 15	n. s. (*p* = 0.84)
Vigorous activity (min/day)	15 (10–24)	17 (11–23)	14 (9–25)	n. s. (*p* = 0.40)
Moderate-to-vigorous activity (min/day)	59 ± 22	57 ± 19	60 ± 24	n. s. (*p* = 0.75)
Total activity (min/day)	306 ± 65	296 ± 58	310 ± 68	(*p* = 0.71)
PAQ-C score	2.92 ± 0.65	2.84 ± 0.65	2.96 ± 0.65	(*p* = 0.49)
Calibrated PAQ tool (%MVPA)	8.05 ± 3.89	7.40 ± 3.02	8.94 ± 2.57	*p* = 0.04

*Data are shown as mean ± SD, or median (IQR). Non-significant results are indicated as n. s*.

Reliability analysis showed a Cronbach's alpha of 0.78 and item-total correlations between 0.22 and 0.45. The raw inter-item correlation coefficients revealed a mean inter-item correlation of 0.35, which varied from 0.04 to 0.60. Two coefficients were below 0.15 (items 2 and 6 and items 4 and 6).

**Figure 1:**
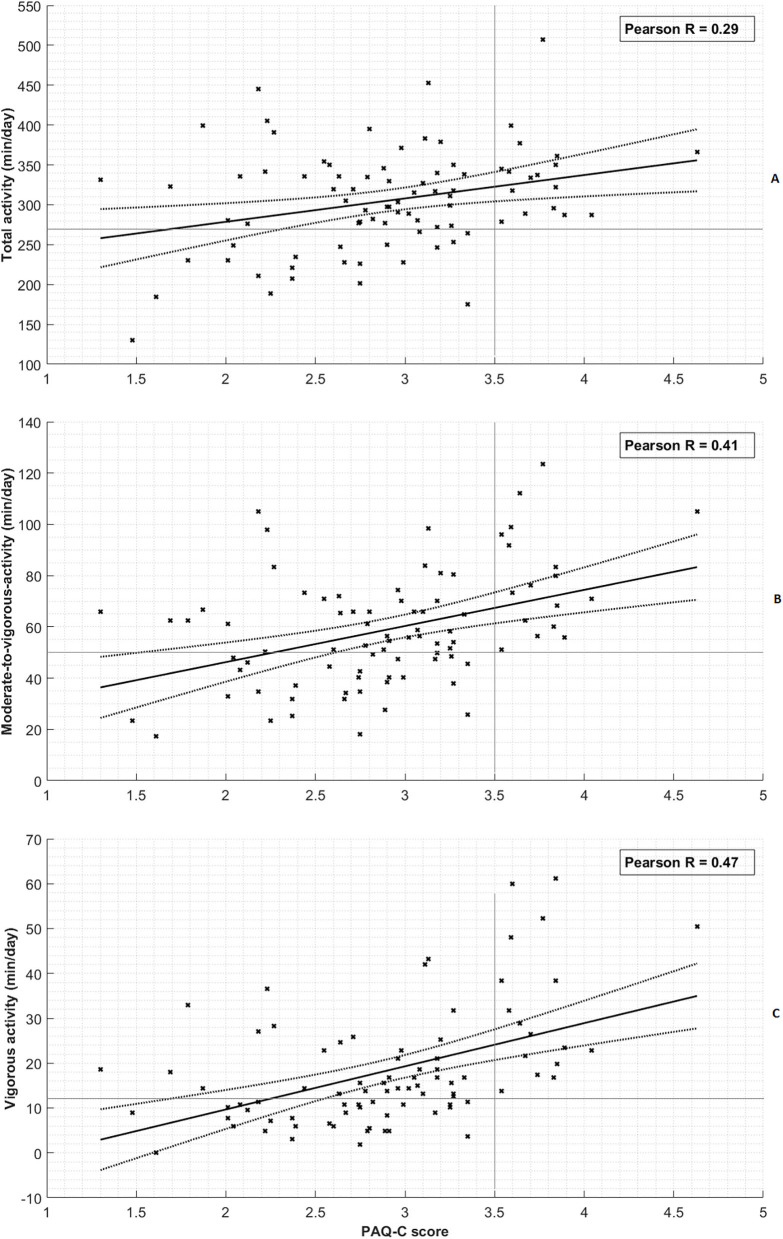
Correlation of physical activity measures with PAQ-C score. **(A)** Total activity. **(B)** Moderate-to-vigorous activity. **(C)** Vigorous activity. Each cross represents a single subject measurement. The thick black line represents the linear regression line and the dashed line denotes its 95% confidence interval. The horizontal and vertical thin gray lines provide an indication of the distinctive character of the PAQ-C threshold of 3.5 for assessing inactivity.

Figure 1 shows the PAQ-C score vs. the activity levels of all children. Figure 1A shows that the PAQ-C scores did weakly correlate with total activity (*R* = 0.29, *p* = 0.007). Figure 1B reveals a moderate correlation for the PAQ-C scores vs. the MVPA (*R* = 0.41, *p* < 0.001). Figure 1C shows a moderate correlation between the PAQ-C scores vs. the vigorous PA (*R* = 0.47, *p* < 0.001). The PAQ-C inversely and weakly correlates to the sedentary activity (*R* = −0.32, *p* = 0.001). Spearman's rho tests with the rounded PAQ-C score showed similar results [total activity (Rho = 0.25, *p* = 0.019), MVPA (Rho = 0.37, *p* < 0.001), vigorous PA (Rho = 0.43, *p* < 0.001), and sedentary PA (Rho = 0.25, *p* = 0.021)].

In 49 children, the PAQ-C score underestimated the amount of MVPA (with a median of 14.0 min and a maximum of 39.5 min). In 36 children, the PAQ-C score overestimated the amount of MVPA (with a median of 16.8 min and a maximum of 56.2 min).

**Figure 2:**
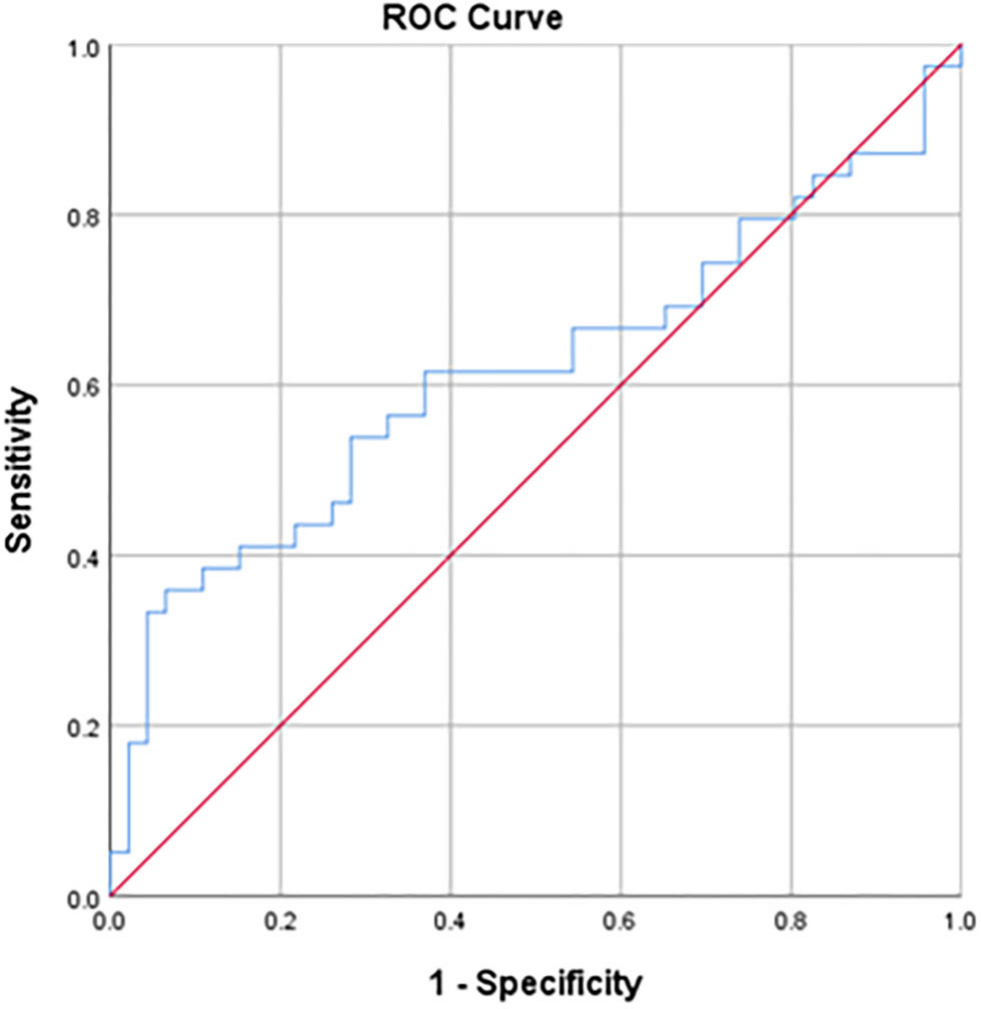
Receiver operator curve of the PAQ-C score and its ability to predict the achievement of daily moderate-to-vigorous physical activity levels (>60 min). The area under the curve is 0.62. The best performance was found with a PAQ-C cutoff of 3.09 (53.8% sensitivity and 71.7% specificity).

The area under the curve (AUC) of the ROC curve was 0.615 (Figure 2). The best performance of the dichotomous test was found with a PAQ-C cutoff of 3.09 (Youden's index = 0.256). With this cutoff, 21 of the 39 children that achieved their daily MVPA level (53.8% sensitivity) and 33 of the 46 children that failed their daily MVPA level (71.7% specificity) were classified correctly (Table 3A). The associated positive and negative predicted values for failing and achieving the daily MVPA goals are 61.8 and 64.7%, respectively. The ROC analysis of the calibrated PAQ tool showed an AUC of 0.587, 69.2% sensitivity, and 52.2% specificity in predicting whether children achieved their daily MVPA level.

**Table 3A:** Classification matrix of the PAQ-C to distinguish the amount of children (*n*) who did and did not achieve the minimum recommended daily MVPA level of 60 min [WHO activity guideline for children (6)].

		**Amount of MVPA**		
		**Achievers** **(>60 min MVPA)**	**Failing** **(<60 min MVPA)**	**Total (*n*)**
PAQ-C score	Score ≥ 3.09	21	13	34
	Score <3.09	18	33	51
	Total (*n*)	39	46	85

Twenty percent (17/85) of the children had a PAQ-C score of 3.5 or higher. None of these children had <50 min of MVPA, indicated by the empty area in the lower right part of Figure 1B. This corresponds to a perfect negative predictive value of 1.0 for assessing physical inactivity (defined as <50 MVPA) with a 3.5 PAQ-C score cutoff (Table 3B).

**Table 3B:** Classification matrix of the PAQ-C threshold of 3.5 to distinguish the amount of children (*n*) above or below a MVPA level of 50 min.

		**Amount of MVPA**		
		**Inactive** **(<50 min MVPA)**	**Active** **(>50 min MVPA)**	**Total (*n*)**
PAQ-C score	Score <3.5	30	38	68
	Score ≥ 3.5	0	17	17
	Total (*n*)	30	55	85

## Discussion

This study showed poor positive and negative predicted values (61.8 and 64.7%, respectively) of the PAQ-C for achieving the recommended daily MVPA level of children. We found a weak association between the PAQ-C score and the total activity in children with asthma and a moderate association between the PAQ-C score and both the MVPA and vigorous PA. However, the results do show that all children with a PAQ-C score above 3.5 performed at least 50 min of MVPA per week. Therefore, these findings indicate a constrained clinical usefulness of the PAQ-C for ruling out physical inactivity in 20% (17/85) of the asthmatic children in this study.

This is the first study that investigated the correlation of the PAQ-C and accelerometry in a pediatric asthma population. In Voss et al. (30), the PAQ-C showed moderate correlations throughout all activity parameters in children with congenital heart disease. Although we found comparable results in the MVPA and the vigorous activities, the correlation with the total activity is considerably lower in our study compared to theirs (*R* = 0.29 vs. *R* = 0.52). This difference might be present due to the fact that many children with asthma adapt their PA pattern. In a previous study, we found that asthmatic children with moderate exercise-induced bronchoconstriction (EIB) tend to perform more light activities compared to children without EIB, while showing similar levels of total activity (due to a reduce in MVPA) (19). The amount of light activities may therefore vary depending on the type and duration of activity and the occurrence of exercise-induced symptoms, explaining the weak correlation with the total activity; hence, the total amount of active minutes primarily consists of minutes in light intense activities.

We identified that a PAQ-Score cut-point of 3.09 discriminates best between those meeting PA guidelines and those that do not. This was comparable compared to previous reported findings in pediatric populations of 2.87 (30). However, the diagnostic value of the PAQ-C for achieving daily MVPA goals was considerably lower in this study compared to Voss et al. (30) (sensitivity of 54 vs. 80%). This discrepancy is assumed to result from the fact that in our asthmatic population, the mean weekly minutes of MVPA was normally distributed and close to the advised 60 min of MVPA, namely, 59 ± 22 min. In the study of Voss et al. (30) the children with congenital heart disease (CHD) showed skewed data with a median of 46 (IQR 31–59) min MVPA, with extreme values of far above the advised 60 min of MVPA. Therefore, the contrast of the amount of MVPA is higher in their population, making the classification of achieving the MVPA goal less prone to misidentification.

The use of the calibrated PAQ tool did not improve the accuracy compared to the self-reported PAQ-C score alone (respectively, AUC of 0.587 compared to 0.615). Although it sounds very plausible to correct the PAQ-C score for age and gender, we did not find evidence that the model of Saint-Maurice et al. (33) could contribute in discriminating children with asthma who meet PA guidelines and children with asthma that do not. Large population studies with accelerometer and self-reported PA documentation should therefore reevaluate the calibrated PAQ-C score model. This moreover enables investigating whether these models have to be corrected for different disease effects (i.e., asthma severity) in order to be applicable in a broader pediatric population.

### Strengths and Limitations

The PAQ-C questionnaire was chosen as best possible questionnaires for evaluating PA. However, the study may be limited as this questionnaire was designed for healthy children. Reliability analysis in this study revealed an acceptable internal consistency (Cronbach's alpha 0.78) for this population and corresponds to similar findings in pediatric studies (13, 25, 29, 36). Moreover, item-total correlations between 0.22 and 0.45 indicated that the items may add reliable variance to the total internal consistency (37). However, raw inter-item correlation coefficients showed that the reliability was not uniform throughout the items. Item 6, questioning the evening activity, deviated the most and showed low correlations. This may be explained by the fact that children's evening activities are more variable, especially in children with asthma as a substantial part of the asthmatic children do not participate in evening sports trainings at all, compared to healthy peers in which almost all children participate in evening sports training. Follow-up research should further look into the possible adaptation of the PAQ-C questionnaire for the pediatric asthma population to increase uniformity and total reliability.

A limitation of this study was that two activity datasets were used with a different methodology for accelerometer-based activity data acquisition. However, the device differences in accuracy of the Actigraph GTM1 and the Actigraph wGT3X-BT were expected to be minor as Vanhelst et al. (38) found both to provide similar classification accuracy. The different wear locations (hip vs. wrist) of the Actigraph devices in both studies might have influenced the classification of activity intensities. However, we minimized this effect by classifying according to the best known practice for each wear location, which is uni-axial cut-points of Evenson et al. (21) for the hip location and tri-axial cut-points of Chandler et al. (20) for the wrist location (39).

A minor limitation of this study was that the baseline characteristics of the two pooled datasets were different in age, BMI *z*-score, and C-ACT score. The participants from the WEARCON study were slightly younger, more overweight, but had better asthma control. However, we believe the total pooled dataset resembles the average asthma population quite well, when compared to other (large) Dutch cohorts (40, 41). Furthermore, no differences were found between the activity parameters, indicating that the total population of the pooled dataset showed similar (variation in) activity patterns. The only exception was the calibrated PAQ-C, which follows from the fact that the participants in the WEARCON study were younger and that the calibrated PAQ-C is corrected for age. This difference did not affect the primary outcomes of this study and therefore allowed the pooling of the data for the aim of this study. By pooling the data, we ensured to have sufficient statistical power for analysis of the relation between the PAQ-C with the accelerometry data.

### Clinical Implication

Although accelerometry is favorable for accurate and objective PA assessment in children with asthma, it seems in this study that the PAQ-C might play a role in pediatric asthma management to rule out physical inactivity in those with a PAQ-C score of 3.5 or higher. The PAQ-C can easily be filled in by asthmatic children before a visit to the outpatient clinic. If the score is equal to or above 3.5, physical inactivity might be excluded, making the PAQ-C a low-cost screening tool contributing to a more efficient care. Still, the group of asthmatic children with a score below 3.5 (80% of the population) need a more precise assessment of their PA levels to identify inactive asthmatic children. This could be achieved with an objective accelerometry assessment.

## Conclusion

This study revealed a weak to moderate relation between subjectively and objectively measured PA. The study furthermore showed poor positive and negative predicted values of the PAQ-C for achieving PA guidelines. However, the results do show that a PAQ-C score of 3.5 or higher might be useful as a low-cost and easy-to-use PA screening tool for ruling out physical inactivity in a portion of the pediatric asthma population.

## Data Availability Statement

The raw data supporting the conclusions of this article will be made available by the authors, without undue reservation.

## Ethics Statement

The studies involving human participants were reviewed and approved by Medical Ethical Committee Twente and Medical Ethical Committee Martini Hospital. Written informed consent to participate in this study was provided by the participants' legal guardian/next of kin.

## Author Contributions

MK, BN, BT, and JD contributed to conception and design of the study. MK, BN, and MT performed the analyses. MK wrote the first draft of the manuscript. MT and AK wrote sections of the manuscript. All authors contributed to manuscript revision, read, and approved the submitted version.

## Funding

The funder had no role in the design of the study or the collection, analysis, and interpretation of the data. We thank the Stichting Pediatrisch Onderzoek Enschede (SPOE) for financially supporting this study.

## Conflict of Interest

The authors declare that the research was conducted in the absence of any commercial or financial relationships that could be construed as a potential conflict of interest.

## Publisher's Note

All claims expressed in this article are solely those of the authors and do not necessarily represent those of their affiliated organizations, or those of the publisher, the editors and the reviewers. Any product that may be evaluated in this article, or claim that may be made by its manufacturer, is not guaranteed or endorsed by the publisher.

## Abbreviations

BMI: Body mass index
C-ACT: Childhood asthma control test
EIB: Exercise-induced bronchoconstriction
FEV_1_: Forced expiratory volume in 1 second
GINA: Global Initiative for Asthma
ICS: Inhaled corticosteroids
IQR: Interquartile range
LABA: Long-acting beta-agonist
MVPA: Moderate-to-vigorous intensity physical activity
PA: Physical activity
PAQ-C: Physical Activity Questionnaire for Children
ROC: Receiver operator characteristic.
